# A new species of the genus *Amphicteis* Grube, 1850 (Annelida, Ampharetidae) from the Yellow Sea, China, together with a redescription of *A. dalmatica* Hutchings & Rainer, 1979

**DOI:** 10.3897/zookeys.988.49934

**Published:** 2020-11-06

**Authors:** Weina Wang, Jixing Sui, Xinzheng Li, Pat Hutchings, João Miguel de Matos Nogueira

**Affiliations:** 1 Department of Marine Organism Taxonomy and Phylogeny, Institute of Oceanology, Chinese Academy of Sciences, 7 Nanhai Road, Qingdao 266071, China Chinese Academy of Sciences Qingdao China; 2 University of Chinese Academy of Sciences, Beijing 100049, China University of Chinese Academy of Sciences Beijing China; 3 Center for Ocean Mega-Science, Chinese Academy of Sciences, Qingdao, China Qingdao National Laboratory for Marine Science and Technology Qingdao China; 4 Laboratory for Marine Biology and Biotechnology, Qingdao National Laboratory for Marine Science and Technology, Qingdao 266000, China Academy of Sciences Qingdao China; 5 Australian Museum Research Institute, Australian Museum, 1 William Street, Sydney, NSW 2010, Australia Australian Museum Sydney Australia; 6 Laboratório de Poliquetologia, Departamento de Zoologia, Instituto de Biociências, Universidade de São Paulo, Rua do Matão, travessa 14, n. 101, São Paulo, 05508-090, Brazil Macquarie University Sydney Australia; 7 Department of Biological Sciences, Macquarie University, North Ryde 2109, Australia. Instituto de Biociências São Paulo Brazil

**Keywords:** *Amphicteis dalmatica*, *Amphicteis hwanghaiensis* sp. nov., taxonomy

## Abstract

A new species of the ampharetid genus *Amphicteis*, *A. hwanghaiensis***sp. nov.**, is described based on material from the Yellow Sea. The new species is characterized by the possession of long, stout, golden paleae with blunt tips, digitiform rudimentary notopodia on the abdominal uncinigers, uncini with a subrostral process, and a narrow rectangular hump separating branchial groups. *Amphicteis dalmatica* was redescribed from type materials at the Australian Museum, Sydney, and the differences between *A. dalmatica* and *A. hwanghaiensis***sp. nov.** are discussed. A key to distinguish *Amphicteis* species described or reported in Western Pacific waters is provided.

## Introduction

Ampharetids are deposit feeders (Jumars et al. 2015) found worldwide, especially at high latitudes and in deep marine environments (Schiaparelli and Jirkov 2016). The genus *Amphicteis* Grube, 1850 is one of the most widely distributed and species-rich genera of the family Ampharetidae (Parapar et al. 2011). Since Schiaparelli and Jirkov (2016) elevated the subspecies of *Amphicteis gunneri* (Sars 1835) (*A. gunneri antarctica* Hessle, 1917, *A. gunneri atlantica* McIntosh, 1885, and *A. gunneri japonica* McIntosh, 1885) to the rank of species, the genus *Amphicteis* encompasses a total of 33 valid species according to WoRMS (Read and Fauchald 2020, as of October 22, 2020). Sui and Li (2017) recently described an additional species (*A. chinensis* Sui & Li, 2017) from China. *Amphicteis* is characterized by the presence of a prostomium with paired longitudinal glandular ridges and transverse or oblique nuchal ridges, four pairs of cirriform branchiae usually arranged in two transverse rows, the presence of paleae, smooth buccal tentacles, 17 thoracic chaetigers with tuberculate ventral cirri on the notopodia, and 13–19 abdominal segments with uncinigerous pinnules and rudimentary notopodia (Day 1964; Fauchald 1977; Holthe 1986; Parapar et al. 2011; Reuscher et al. 2015). Five species of *Amphicteis* were previously described or reported in Chinese waters (Sun 1990; Huang 1994; Liu 2008; Sui and Li 2017): *A. gunneri*; *A. glabra* Moore, 1905; *A. scaphobranchiata* Moore, 1906; *A. mederi* Annenkova, 1929, and *A. chinensis*. Sui and Li (2014) examined all of the specimens deposited in the Marine Biological Museum of the Chinese Academy of Sciences and found that the record of *A. mederi* in China (Sun 1990) was a misidentification of animals belonging to another genus, described therein as *Paramphicteis sinensis* Sui & Li, 2014.

*Amphicteis gunneri*, the type species of the genus, has previously been considered as a cosmopolitan species until Hartley (1985) redescribed the type specimen held at the Zoological Museum, University of Oslo, and concluded that the cosmopolitan status of *A. gunneri* was unjustified. Parapar et al. (2011) suggested that the true distribution of that species may be restricted to the Arctic and North Atlantic European waters, with a southern boundary at the English Channel. They also proposed that until a global revision of *Amphicteis* was completed, the specific name *A. gunneri* should be used with caution.

During recent biodiversity surveys in the Yellow Sea of China, two specimens were collected and confirmed as belonging to an undescribed species of *Amphicteis*. We herein describe these specimens as new to science.

In order to support the “new species” status of our specimens, we also redescribe herein *A. dalmatica* Hutchings & Rainer, 1979, from southeastern Australia, which had not been discussed by Schiaparelli and Jirkov (2016) as belonging to the “blunt stout paleae” group of species, as defined by those authors. Finally, we include a key to the identification of all species of *Amphicteis* occurring in Western Pacific.

## Materials and methods

The two specimens were collected using a 1.5 × 0.5 m Agassiz trawl from the southern Yellow Sea of China in November 2019. Specimens were preserved in 75% ethanol, then deposited in the Marine Biological Museum of the Chinese Academy of Sciences. The specimens were photographed with a digital camera attached to a Nikon AZ100 microscope and drawn with a camera lucida attached to a Nikon SMZ1500 microscope.

The type material of *A. dalmatica* was examined at the Australian Museum, Sydney. The animals were studied using a stereomicroscope, and one paratype was photographed. Notopodia and neuropodia were removed from different regions of the body, mounted on slides with polyvinyl lactophenol (PVLP), and examined and photographed using compound microscopes. For SEM examination, another paratype was dehydrated in an ethanol series, then critical-point dried, sputter coated with gold, and examined at the SEM Laboratory, AM. Photos using stereo and compound microscopes were also taken at the SEM Laboratory, AM. All photos were edited with Adobe Photoshop CC software.

## Systematics

### Family Ampharetidae Malmgren, 1866

#### Genus Amphicteis

Taxon classificationAnimaliaTerebellidaAmpharetidae

Grube, 1850

0F1B06FF-1903-527F-81CC-F788AFE13FCB

##### Type species.

*Amphitrite gunneri* Sars, 1835

##### Diagnosis.

Prostomium with middle lobes as paired longitudinal glandular ridges and nuchal organs as transverse or oblique nuchal ridges. Buccal tentacles usually smooth. Four pairs of cirriform branchiae usually arranged in two transverse rows. Notochaetae in segment II present and usually developed as strong paleae. Seventeen thoracic chaetigers from segment III with notopodia bearing tuberculate ventral cirri. Modiﬁed notopodia and intermediate uncinigers absent. Fourteen thoracic uncinigers with uncini-bearing neuropodial tori (usually with small dorsal papilla) from segment VI. Usually 15 abdominal uncinigers present. Abdominal uncinigers with rudimentary notopodia and uncini-bearing pinnules with digitiform or cirriform dorsal cirri. Usually one pair of anal cirri present, inserted laterally on pygidium. Thoracic and abdominal uncini with a single row of teeth.

#### Amphicteis hwanghaiensis sp. nov.

Taxon classificationAnimaliaTerebellidaAmpharetidae

51D640FC-6489-59EF-BD70-6B7A5F1F6EF9

http://zoobank.org/9AF8951F-61CE-406C-982F-9095865B8D04

Figs 1 , 2 , 3 , 4

##### Material examined.

***Type material*.** Yellow Sea, China (33°58.45'N, 123°57.02'E; 77 m deep), subtidal in mud, collected 28 November 2019. ***Holotype***: MBM286623; ***Paratype***: MBM286624, 1 specimen.

##### Description.

**Figure 1.**
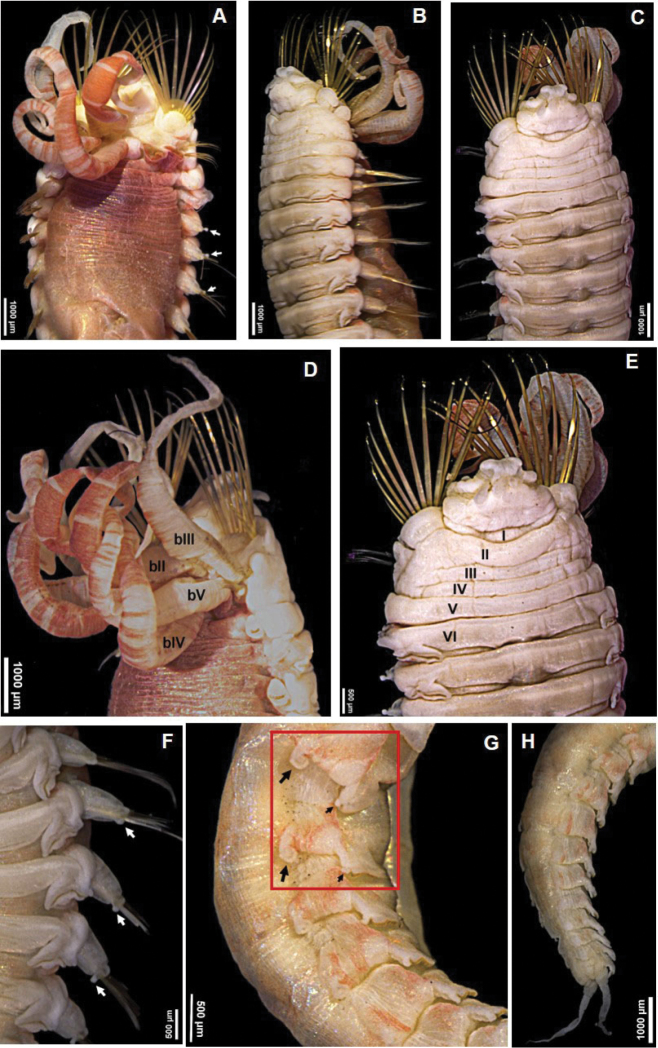
*Amphicteis hwanghaiensis* sp. nov. (holotype) **A** anterior end, dorsal view **B** anterior end, lateral view **C** anterior end, ventral view **D** branchiae **E** anterior end **F** thoracic parapodia, arrows point to notopodial cirri **G** abdominal parapodia, arrows point to notopodial (large) and neuropodial (small) cirri **H** posterior end, ventral view. Numbers refer to segments; ll = lower lip, bII–V = branchiae, segments II–V, respectively.

**Figure 2.**
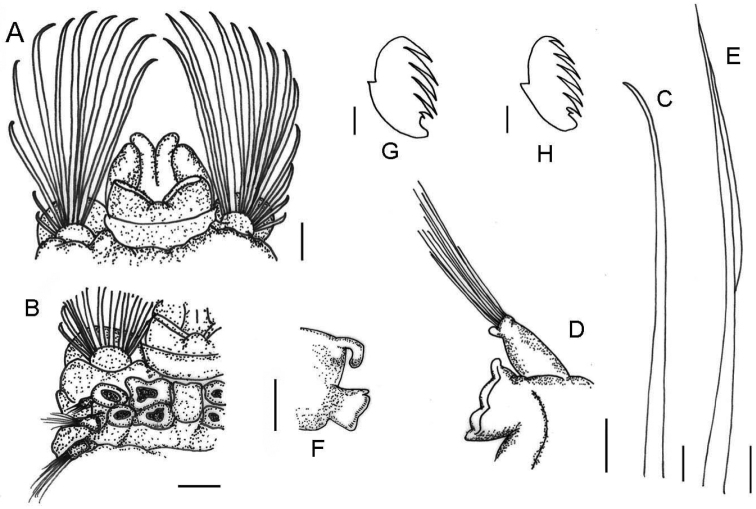
*Amphicteis hwanghaiensis* sp. nov. (holotype) **A** prostomium, dorsal view **B** position of branchiae, dorsal view **C** paleae **D** thoracic parapodium **E** limbate capillary notochaeta **F** abdominal parapodium **G** thoracic uncinus **H** abdominal uncinus. Scale bars: 500 µm (**A, B, D, F**); 250 µm (**C**); 100 µm (**E**); 10 µm (**G, H**).

**Figure 3.**
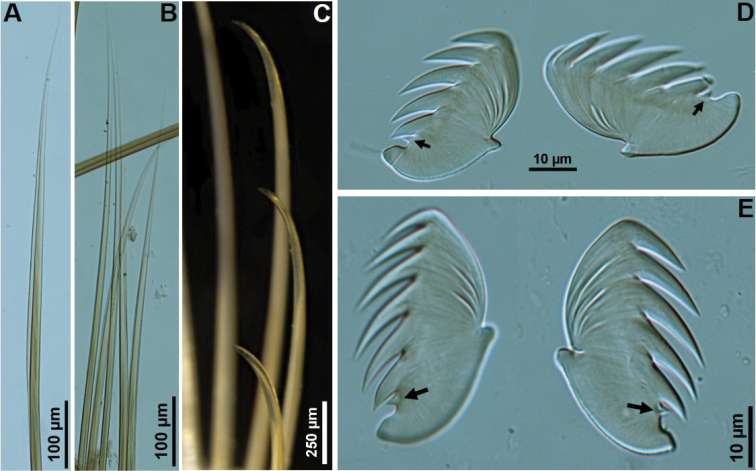
*Amphicteis hwanghaiensis* sp. nov. (holotype) **A** limbate capillary notochaeta **B** notochaetae **C** paleae **D** thoracic uncini, arrows point to subrostal process **E** abdominal uncini, arrows point to subrostal process.

***Holotype*** Complete, length 27.8 mm, thoracic width 5.5 mm. Dorsum of thoracic segments and branchiae with red pigmentation (Fig. 1A, D). Thorax and abdomen well defined; thorax approximately twice as wide and long as abdomen; barely tapering towards posterior part. Prostomium with middle lobe as paired longitudinal glandular ridges, slightly diverging distally, V-shaped, gap between glandular ridges absent (Fig. 2A); eyespots absent. Nuchal organs as paired nuchal ridges separated by a small median gap, V-shaped (Fig. 2A). Segment I inconspicuous, barely visible laterally, in superior view. Segment II developed ventrally and laterally, bearing paleae, covered by branchiae dorsally (Figs 1A–E, 2A, B). Four pairs of long and tapering branchiae, in 2 transverse rows on segments III and IV, separated by a mid-dorsal rectangular hump of half inner branchiae width (Figs 1A, D, 2B); inner branchiae 2 times thicker than outer ones; innermost branchiae of anterior transverse row originating from segment II, outermost branchiae of anterior transverse row originating from segment III, innermost branchiae of posterior transverse row originating from segment IV, outermost branchiae of posterior transverse row originating from segment V (Fig. 2B). Left and right groups of golden paleae present on segment II with 11 on right side and 13 on left side (Figs 1A–E, 2A). Paleae arranged in shallow arcs with the longest paleae innermost; stout and slightly curved dorsally and tapering to short blunt tips; well developed, twice as long as prostomium (Figs 1A–E, 2A, C, 3C). Notopodia with capillary chaetae and tuberculate ventral cirrus from segment III, present on 17 chaetigers (Figs 1F, 2B, D, E, 3A, B); anterior notopodia small, increasing in size from ﬁrst to fourth pair (Fig. 1A–C). Neuropodial tori with uncini from segment VI, present on 14 thoracic uncinigers; tori without offset dorsal lobe (Figs 1F, 2D). Continuous ventral shields present to approximately thoracic unciniger 12. Elevated or modiﬁed notopodia absent. Intermediate uncinigers absent. Fifteen abdominal uncinigers with digitiform rudimentary notopodia (Figs 1G, 2F). Pinnules with tiny tuberculate dorsal cirrus (Figs 1G, 2F). Thoracic and abdominal uncini arranged in single vertical rows with subrostral process and five or six teeth in a single row over basal prow (Figs 2G, H, 3D, E). Pygidium with terminal anus and two laterally attached tapering anal cirri, approximately as long as the last five chaetigers (Fig. 1H).

**Figure 4.**
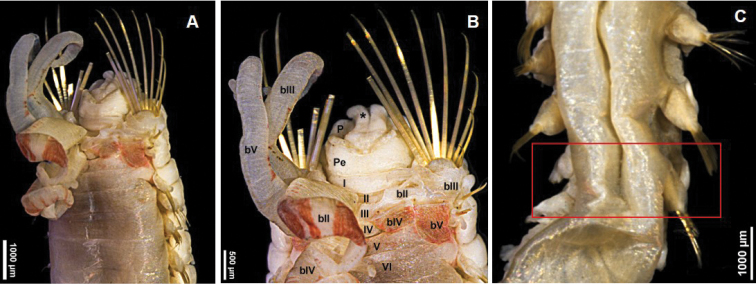
*Amphicteis hwanghaiensis* sp. nov. (paratype) **A** anterior end, dorsal view **B** anterior end **C** thoracic parapodia. Numbers refer to segments; bII–V = branchiae, segments II–V, respectively; P = prostomium; * = middle lobe of prostomium; Pe = peristomium.

***Paratype*** complete, 31 mm long, 4.5 mm wide, with ten paleae on right side and eight on left (Fig. 4A, B). Eighteen thoracic chaetigers one side and 17 thoracic chaetigers on the other side (Fig. 4C). Dorsum of thoracic segments shows no pigmentation and only inner branchiae have several red bands.

##### Etymology.

The species is named after its type locality in the Yellow Sea of China. The species name is an adjective in the nominative singular, derived from “hwanghai” which means “Yellow Sea” in Chinese, with the Latin suffix -ensis to denote a place.

##### Distribution.

Yellow Sea at 79 m depth.

##### Remarks.

The presence of stout paleae with blunt tips is characteristic for *A. hwanghaiensis* sp. nov.. Schiaparelli and Jirkov (2016) provided a revision of the genus *Amphicteis* and concluded that out of the 38 *Amphicteis* species (known at that time), only five species have this type of paleae: *A. mederi*, *A. midas* (Gosse, 1855), *A. taurus* Reuscher, Fiege & Imajima, 2015, *A. ninonae* Jirkov, 1985, and *A. teresae* Schiaparelli & Jirkov, 2016. According to Reuscher et al. (2015), *A. dalmatica* Hutchings & Rainer, 1979 and *A. philippinarum* Grube, 1878 also have short and poorly developed paleae. The latter species differs from *A. hwanghaiensis* sp. nov. by having foliose branchiae and uncini without a subrostral process, while *A. hwanghaiensis* sp. nov. only have cirriform branchiae and uncini with a subrostral process. To make clear the distinction between members of *A. dalmatica* and our new species, the type material of *A. dalmatica* was examined, redescribed, and compared with the new species (below).

All the other *Amphicteis* species have paleae with fine filamentous tips; the difference between fine-tipped and stout-tipped paleae is easy to distinguish. According to Schiaparelli and Jirkov (2016), the shape of blunt, stout paleae from the five known species belonging to this group are all very similar, but there are other diagnostic morphological differences, which can be used to distinguish them from the new species.The difference between *A. mederi* and *A. hwanghaiensis* sp. nov. is that *A. mederi* has abdominal pinnules with a cirriform dorsal cirrus, while the new species has a tuberculate dorsal cirrus; the thoracic and abdominal uncini of *A. hwanghaiensis* sp. nov. have five or six teeth in a single row over the basal prow while the uncini in *A. mederi* have six teeth (Annenkova 1929; Uschakov 1955). According to Schiaparelli and Jirkov (2016), who checked the holotype of *A. mederi*, the prostomial glandular ridges of *A. mederi* are separated by a wide median gap equal to the width of the ridge, while a gap between glandular ridges is absent in *A. hwanghaiensis* sp. nov. (Fig. 2A).

A comparison of *A. midas* and *A. hwanghaiensis* sp. nov. shows differences in the rounded spots on the anterior dorsum and the dark transversal pigment bands on its branchiae (Schiaparelli and Jirkov 2016); in contrast, the new species has red pigmentation on its branchiae. In addition, the area between the branchial groups is very different. *Amphicteis hwanghaiensis* sp. nov. has a narrow mid-dorsal rectangular hump between the inner branchiae while the area between the branchial groups of *A. midas* is flat and unmodified (Hartley 1985).

*Amphicteis taurus* is clearly distinct and differs from *A. hwanghaiensis* sp. nov. in the following features. The paleae of *A. taurus* are unique in the genus *Amphicteis*, being strongly enlarged, nearly straight with a uniform thickness over the entire length, and tips rounded, at about a 45-degree angle to the body. *Amphicteis taurus* is also different from *A. hwanghaiensis* sp. nov. by the smaller prostomial glandular ridges and the wide gap separating them. Other differences between them are the longer, annulated cephalic region (peristomium and possibly segment I) of *A. taurus* and the shorter cephalic region of the new species (Reuscher et al. 2015).

According to original description, *A. teresae* has a larger number of paleal chaetae (15–17 on each side). *Amphicteis hwanghaiensis* sp. nov. has a lower lip with a narrow, distinct, and white middle transversal band which is absent in *A. teresae*. Uncini of *A. hwanghaiensis* sp. nov. have ﬁve or six teeth besides the subrostral tooth, while uncini of *A. teresae* usually have ﬁve. As for eyespots, which are absent in new species, Schiaparelli and Jirkov (2016: 541) said that “Another clear character of *Amphicteis teresae* sp. n. that distinguishes it from the other related ones having blunt paleal chaetae is the presence of an eyespot”. Furthermore, *A. teresae* is found in Antarctica.

*Amphicteis ninonae*, recorded from Norwegian Sea and Arctic Seas, is most similar to the new species; however, members of this species are distinguished because, according to Jirkov (1985), the paleae are dark brown, while those of *A. hwanghaiensis* sp. nov. are golden. *Amphicteis hwanghaiensis* sp. nov. also has a narrow rectangular hump between the branchial groups, while the area between the branchial groups of *A. ninonae* is flat and unmodified. Parapar et al. (2011) also suggested that *A. ninonae* seems to be restricted to the north and east coasts of Iceland.

Four species of *Amphicteis*, *A. glabra*, *A. gunneri*, *A. scaphobranchiata*, and *A. chinensis*, have been recorded from Chinese seas, according to Sui and Li (2017). The new species differs from these species by having blunt and stout tipped paleae, as these four species belong to the group of *Amphicteis* species with tips of paleae sharply tapering into fine ﬁlaments.

Among the species from the Western Paciﬁc, specimens belonging to *Amphicteis malayensis* Caullery, 1944 differ from those of *A. hwanghaiensis* sp. nov. by the possession of prostomial eyespots and a wide median gap between glandular ridges. Members of *A. theeli* Caullery, 1944 and *A. quadridentata* Caullery, 1944 have 14 and 16 abdominal uncinigers, respectively. The branchiae of individuals of *A. spinosa* Reuscher, Fiege & Imajima, 2015 have four rows of pointed protuberances, while branchiae are smooth among specimens belonging to our new species. Finally, specimens belonging to *A. uncopalea* Chamberlin, 1919, found in the North-western Paciﬁc, have well-developed paleae with curly and fine tips, and a distinct rounded lobe behind the paleae originating from segment III.

#### Amphicteis dalmatica

Taxon classificationAnimaliaTerebellidaAmpharetidae

Hutchings & Rainier, 1979

EE2CA4DF-4617-52B4-BA3E-A0954A891380

Figs 5 , 6 , 7

Amphicteis dalmatica Hutchings and Rainer 1979: 783–786, fig. 9 A–E.

##### Material examined.

***Type material*.** Australia, New South Wales, Pittwater, found in *Zostera* or *Posidonia* beds, 1–4 m deep. ***Holotype***: AM W.8672, incomplete, 7 mm long, 2 mm wide anteriorly. ***Paratypes***: AM W.8230, W.8242, W.8243, W.8249, W.8251, W.8252, W.8253, W.11667, W.11668, all incomplete; complete paratypes W.8243, W.8252, W.11667; W.8230 (only specimen with upper lip and buccal tentacles exposed) mounted for SEM examination.

##### Redescription.

**Figure 5.**
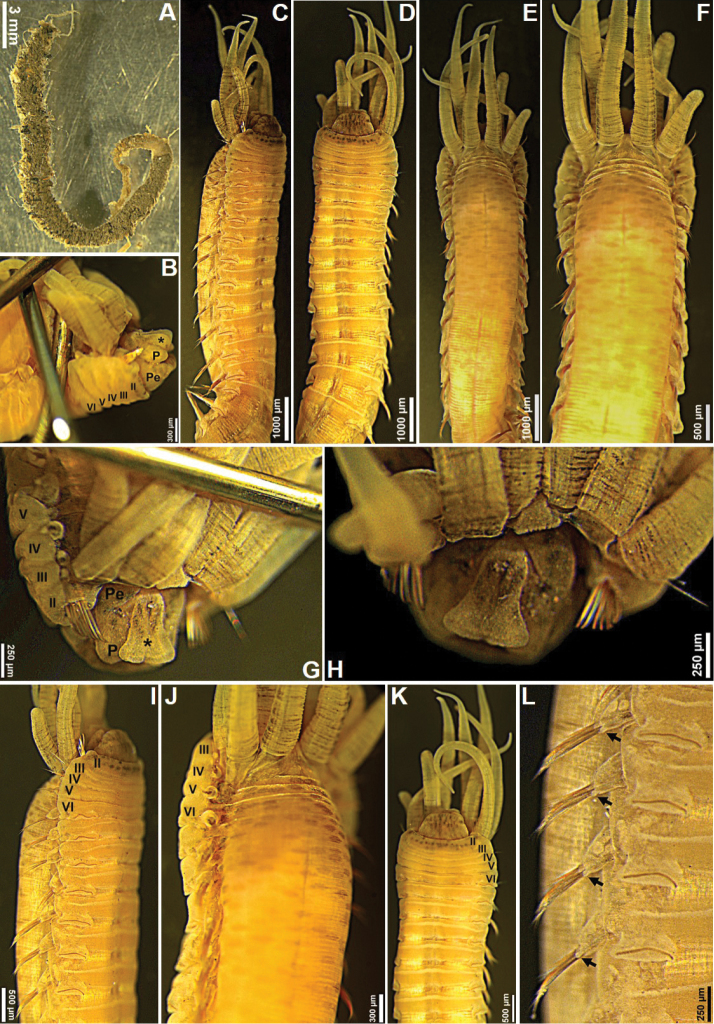
*Amphicteis dalmatica* (paratype, AM W.11667) **A** tube **B** anterior end, right lateral view **C** anterior body, ventrolateral view **D** anterior body, ventral view **E** anterior body, dorsal view **F** closer view of the anterior body, dorsal view **G, H** prostomium and anterior most segments, dorsolateral and dorsal views, respectively **I** anterior body, ventrolateral view **J** anterior body, dorsolateral view **K** anterior body, ventral view **L** thoracic parapodia, arrows point to notopodial cirri. Numbers refer to segments; ll = lower lip; P = prostomial lobes; Pe = peristomium.

**Figure 6.**
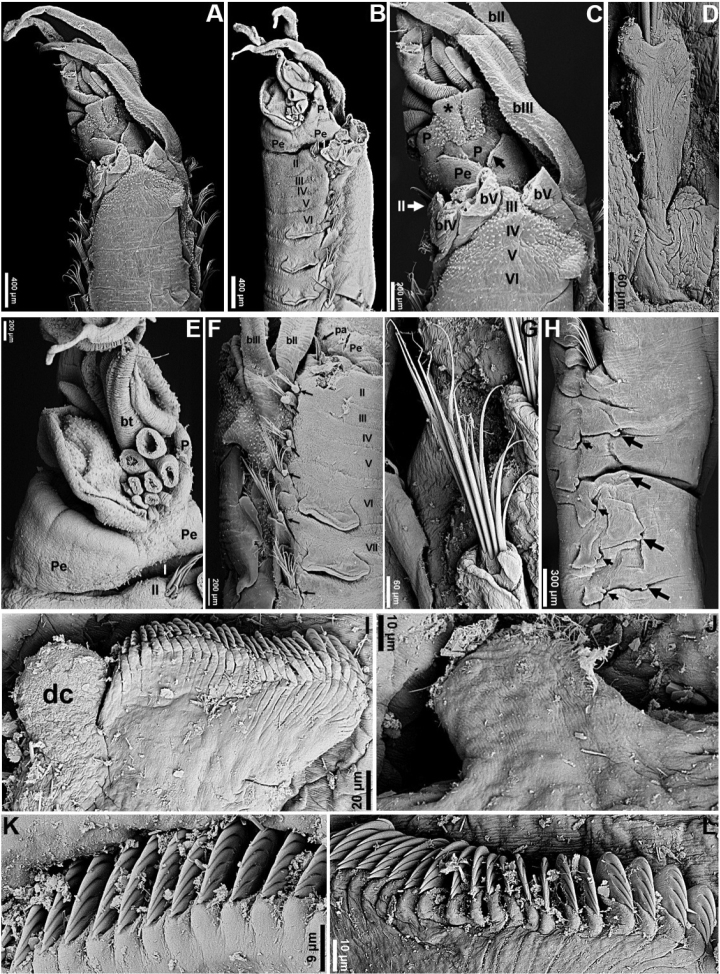
*Amphicteis dalmatica* (paratype, AM W.8230) **A** anterior body, dorsal view **B** anterior body, left lateral view **C** closer view of the anterior body, dorsal view **D** notopodium, segment 6 **E** peristomium and anteriormost segments, ventrolateral view **F** anteriormost segments, lateral view, arrows point to notopodial cirri **G** notochaetae, segment 6 **H** transition between thorax and abdomen, small arrows point to dorsal neuropodial cirri, large arrows point to notopodial cirri **I** abdominal neuropodium **J** abdominal notopodial cirrus **K** uncini, segment 8 **L** abdominal uncini. Numbers refer to segments; bII–V = branchiae, segments II–V, respectively; bt = buccal tentacles; ll = lower lip; P = prostomium; * = middle lobe of prostomium; Pe = peristomium; ul = upper lip.

**Figure 7.**
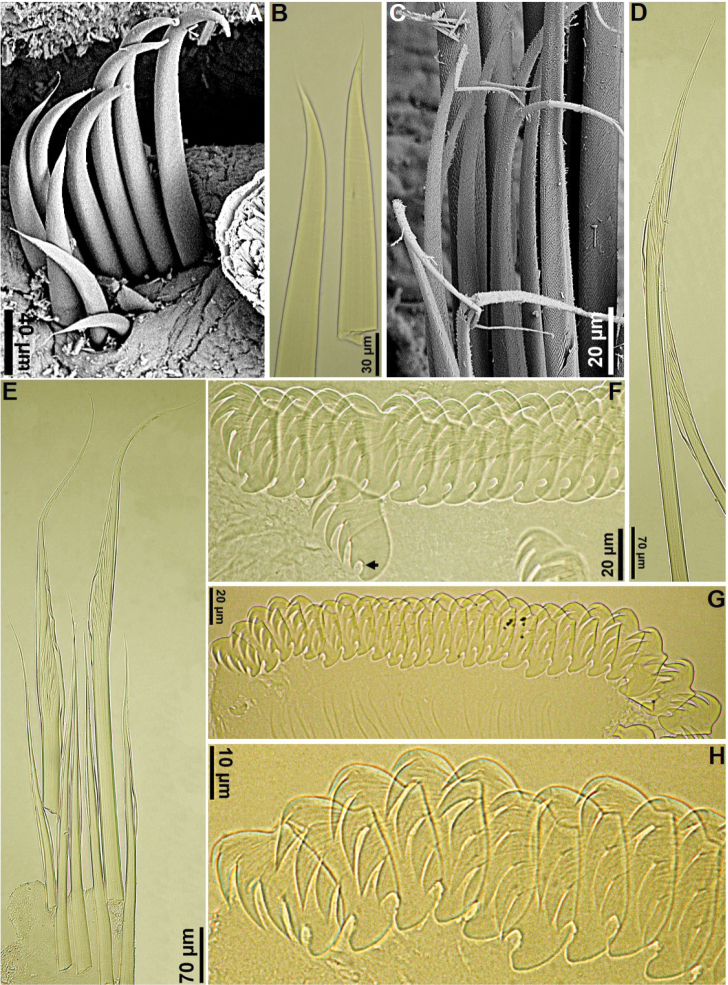
*Amphicteis dalmatica* (paratype, AM W.8230) **A, B** paleae **C** detail of notochaetae, segment 7 **D** chaetae from longest row of notochaetae, segment 14 **E** notochaetae, segment 14 **F** uncini, segment 14, arrow points to subrostal process **G, H** abdominal uncini.

Types with dorsum of anterior segments speckled with pigmented spots, pigmentation decreasing posteriorly. Pigmentation still visible after decades of storage in alcohol, although paler than when originally described (compare our Fig. 5 with fig. 9 in the original description, especially the branchial pigmentation, most of which has been lost over time). Mucous tube with embedded sand and shell particles (Fig. 5A). Prostomium well developed, with a mid-dorsal trilobed process (Figs 5B, G, H, 6A–C), middle lobe as paired longitudinal glandular ridges, wider distally, T-shaped, gap between glandular ridges absent (Figs 5B, G, H, 6A–C); lateral lobes each with a cluster of eyespots basally. Nuchal organs as paired nuchal ridges, touching each other basally, V-shaped, lacking median gap; buccal tentacles smooth (Figs 5B–E, G–I, K, 6A–C, E, F). Segment I inconspicuous, barely visible laterally, in superior view (Fig. 6B, E); segment II developed ventrally and laterally, bearing paleae, covered by branchiae dorsally (Figs 5B–D, G–I, K, 6A–C, E, F). Four pairs of long and tapering branchiae, arising free from body wall in 2 transverse rows, on segments III and IV, each with 1 long and thick filament on each side, separated in left and right groups by a mid-dorsal triangular hump (Figs 5B–K, 6A–C). All branchial filaments about same size, originating from segments II–V, arising as free filaments from segments III and IV, 2 pairs on each segment, in transverse rows, as follows: on segment III, outer pair originating from segment II, inner pair originating from segment III; on segment IV, outer pair originating from segment IV, inner pair from segment V (Figs 5B–K, 6A–C, F). Segment II with ~10 short stout notopodial paleae on each side, arranged in shallow arcs, paleae distally pointed, with short filiform tip, frequently broken off; paleae remarkably small, about same size as notochaetae, but stouter (Figs 5B, C, G–I, K, 6B, E, F, 7A, B). Notopodia with capillary chaetae starting from segment III and extending through 17 chaetigers; notopodia each with a tuberculate ventral cirrus (Figs 5L, 6D, F, G, 7C–E); first 3 pairs aligned laterally to following pairs and increasing progressively in size, all 3 much shorter than those from segment VI onwards (Figs 5B, C, E–G, I, J, 6A–C, F). Neuropodial tori with uncini from segment VI, present in 14 thoracic uncinigers; tori as raised trapezoidal structures throughout, larger on thorax (Figs 5C, D, I, L, 6B, F). Continuous ventral shields present to approximately thoracic unciniger 12 (Fig. 5C, D, I, K). Elevated or modiﬁed notopodia absent. Intermediate uncinigers absent. Fifteen abdominal uncinigers with tuberculate rudimentary notopodia (Fig. 6H, J). Pinnules with tiny tuberculate dorsal neuropodial process (Fig. 6H, I). Thoracic and abdominal uncini arranged in single vertical rows with barely conspicuous subrostral process and five or six teeth in a single row over basal rounded prow; thoracic uncini with teeth progressively increasing in size until fourth, fifth (distal) tooth shorter (Figs 6K, 7F); abdominal uncini with sixth tooth, when present, much shorter than other teeth (Figs 6L, 7G, H). Pygidium with one pair of long, gently tapering anal cirri.

##### Distribution.

New South Wales, Australia.

##### Remarks.

Members of *A. dalmatica* are clearly distinct and differ from our new species in the following features. In *A. dalmatica*, the paleae are poorly developed, prostomial lateral lobes each have a cluster of eyespots basally, uncini have barely conspicuous subrostral process, and a distinct spotted pigmentation pattern is present on the dorsum of thoracic segments and branchiae. Members of *Amphicteis hwanghaiensis* sp. nov. have well-developed paleae that are twice as long as the prostomium. Uncini of *A. hwanghaiensis* sp. nov. have a much larger subrostral process. Furthermore, the type locality of *A. dalmatica* is New South Wales, Australia, in seagrass beds.

#### Key to *Amphicteis* species from the Western Pacific

**Table:** 

1	Paleae with blunt tips	**2**
–	Paleae with fine tips	**5**
2	Paleae poorly developed, not exceeding the prostomium	***A. dalmatica***
–	Paleae well developed, two times longer than the prostomium	**3**
3	Paleae straight	***A. taurus***
–	Paleae with curly tips	**4**
4	Wide median gap between glandular ridges	***A. mederi***
–	Gap between glandular ridges absent	***A. hwanghaiensis* sp. nov.**
5	16 abdominal uncinigerous segments	**6**
–	Fewer than 16 abdominal uncinigerous segments	**7**
6	Rounded lobe originating from segment III behind the paleae	***A. chinensis***
–	Lobe absent behind the paleae	***A. quadridentata***
7	14 abdominal uncinigers	***A. theeli***
–	15 abdominal uncinigers	**8**
8	Paleae poorly developed, not exceeding the prostomium	***A. philippinarum***
–	Paleae well developed, exceeding the prostomium	**9**
9	Branchiae with four rows of pointed protuberances	***A. spinosa***
–	Branchiae smooth, without pointed protuberances	**10**
10	At least one pair of foliaceous branchiae	**11**
–	All branchiae cylindrical	**13**
11	One pair of foliaceous branchiae	**12**
–	Two pairs of foliaceous branchiae	***A. bifolium***
12	All abdominal uncini with teeth in one row	***A. nikiti***
–	Some abdominal uncini with several rows of teeth	***A. scaphobranchiata***
13	Rrounded lobe behind the paleae that originates from segment III	***A. uncopalea***
–	No lobe behind the paleae	**14**
14	Nuchal organs with four lobes and many pigment spots	***A. malayensis***
–	Nuchal organs smooth without pigment spots	**15**
15	Paleae 8–10 on each side	***A. glabra***
–	Paleae up to 20 on each side	***A. gunneri***

## Supplementary Material

XML Treatment for Amphicteis

XML Treatment for Amphicteis hwanghaiensis

XML Treatment for Amphicteis dalmatica
